# Optimal Strategies for Control of COVID-19: A Mathematical Perspective

**DOI:** 10.1155/2020/4676274

**Published:** 2020-11-28

**Authors:** Baba Seidu

**Affiliations:** Department of Mathematics, Faculty of Mathematical Sciences, C. K. Tedam University of Technology and Applied Sciences, Navrongo, Ghana

## Abstract

A deterministic ordinary differential equation model for SARS-CoV-2 is developed and analysed, taking into account the role of exposed, mildly symptomatic, and severely symptomatic persons in the spread of the disease. It is shown that in the absence of infective immigrants, the model has a locally asymptotically stable disease-free equilibrium whenever the basic reproduction number is below unity. In the absence of immigration of infective persons, the disease can be eradicated whenever ℛ_0_ < 1. Specifically, if the controls *u*_*i*_,  *i*=1,2,3,4, are implemented to 100% efficiency, the disease dies away easily. It is shown that border closure (or at least screening) is indispensable in the fight against the spread of SARS-CoV-2. Simulation of optimal control of the model suggests that the most cost-effective strategy to combat SARS-CoV-2 is to reduce contact through use of nose masks and physical distancing.

## 1. Introduction

Starting in November 2019, from the city of Wuhan, China, a disease caused by a novel coronavirus (SARS-CoV-2) has ravaged the entire world, causing the World Health Organization (WHO) to declare it as a pandemic of international concern. As of 8 June 2020, the virus had affected about 188 countries and regions, resulting in over 7 million infections and over 400,000 deaths globally [1]. During talking, coughing, or sneezing by infected persons, the virus is released through droplets and can be inhaled by susceptibles who are in close contact. The virus may also be picked up by susceptible persons from surfaces that got contaminated by droplets from infected persons and infection may result if the susceptible touches their face with the contaminated hands or objects. Symptoms of infection usually appear between 2 and 14 days and may include fever or chills, cough, shortness of breath or difficulty breathing, fatigue, muscle or body aches, headache, new loss of taste or smell, sore throat, congestion or runny nose, nausea or vomiting, and diarrhoea. When infected persons have trouble breathing, persistent pain or pressure in the chest, new confusion, inability to wake up or stay awake, and/or bluish lips or face, then it is time to seek emergency treatment. With no known proven vaccine and treatment drug so far, nonpharmaceutical interventions including use of nose masks, hand washing (sanitizing), and other safe health protocols are a major part in the fight against the infection. Mathematical modelling has proven to be very helpful in increasing the understanding of the spread and providing optimal strategies towards controlling infectious diseases (see [2] and reference therein). As a result, a number of mathematical models have been proposed to study the spread of SARS-CoV-2 and to provide direction towards control (see [3, 4] and the references therein). Specifically, [5] developed a mathematical model to study the impact of nonpharmaceutical interventions on the spread of COVID-19, concluding that the use of face masks and adhering to social distancing are key in the fight against the disease. The model in [6, 7] proposed an optimal control problem that sought to advise what governments could do to curb COVID-19 spread. We note that the majority of those infected with COVID-19 are asymptomatic, and if they are not tested, they may spread the virus without knowing. Thus, the role of symptomatic infected persons in the spread of COVID-19 needs to be studied properly. Use of nose masks and/or face shields, social/physical distancing, and disinfection of surfaces are some nonpharmaceutical interventions that have been proposed to help curb COVID-19. However, since the implementation of these controls comes at some cost, the need to determine the best combination of these control cannot be overemphasized. To the best of our knowledge, no work has been done considering optimal control of COVID-19 in the presence of infectious asymptomatic persons. With better strategies needed to curb the disease, an optimal control problem with four controls (namely, use of face masks and social distancing *u*_1_, avoidance of touching contact surfaces *u*_2_, prevention of surface contamination *u*_3_, and disinfection of environment *u*_4_) is proposed, and the cost-effectiveness of all sixteen possible combinations of these controls is computed.

## 2. The COVID-19 Model

Given a population of the time-dependent size of *N*(*t*) that is subdivided into susceptibles, *S*(*t*), asymptomatically infected, *E*(*t*), clinically infected (those with mild symptoms *I*_1_(*t*) and those with severe symptoms *I*_1_(*t*)), and the recovered, *R*(*t*), so that *N*=*S*+*E*+*I*_1_+*I*_2_+*R*, and denoting the concentration of coronavirus on surfaces by *V*(*t*), a mathematical model to describe the spread of coronavirus in such a population is constructed.

Even though governments around the world have sought to control spread by employing several measures, among which is the restriction of immigration, this measure is not very effective in most African countries due to ineffective border control and use of unapproved routes. This explains how the Northern region of Ghana recorded its first cases, who were all immigrants from Guinea that were undetected until they were in Tamale. We assume that the population is increased due to immigrants at rate Λ with proportions *f*_1_, *f*_2_ being asymptomatic and infected with mild symptoms, respectively, and the remainder being susceptible. Susceptible individuals get infected due to effective contact with infectious (*E*, *I*_1_, and *I*_2_) at rate *λ*_1_=*βc*(1 − *u*_1_)(*η*_1_*E*+*I*_1_+*η*_2_*I*_2_)/(*N* − *q*(*E*+*I*_1_+*I*_2_)) where *η*_1_ > 1 and *η*_2_ ≪ 1 are modification factors accounting for increased (reduced) infectivity of exposed persons and those with severe symptoms. The parameter *q* accounts for the effectiveness of self-quarantine of exposed and mildly symptomatic persons and hospitalization/isolation of severely symptomatic persons in curbing the spread of COVID-19. The sad reality is that most of the infected persons are asymptomatic but are infectious and, therefore, pose a greater threat as they may continue to infect people around them without even knowing. Those with severe symptoms are often at isolation centres where utmost care is taken by the healthcare workers who nurse them, leading to reduced infectivity. The parameters *β* and *c* are, respectively, the transmission probability and the average number of contacts of the infected per day. The parameter 0 ≤ *u*_1_ ≤ 1 is taken to be physical distancing control, such that *u*_1_=1 and *u*_1_=0, respectively, represent perfect observation of preventive protocols (like physical distancing, hand washing, and sanitizing) and noncompliance with the physical distancing and other preventive protocols. It has also been found that the infected persons may deposit the virus on surfaces which can stay for up to 72 hours [8] and may be picked up by susceptibles at rate *λ*_2_=*β*_*v*_(1 − *u*_2_)*V*/(*K*+*V*) (Michaelis-Menten functional response) where *K* is the half-saturation constant of the coronaviruses in the environment, *β*_*v*_ is the rate of uptake of coronaviruses from coronavirus-infected surfaces, and 0 ≤ *u*_2_ ≤ 1 is a control measure accounting for avoidance of touching of infected surfaces and/or washing of hands. Carriers of the virus are assumed to shed the virus unto surfaces at rate *ξ* and that process is impeded by way of controls 0 ≤ *u*_3_ ≤ 1 including proper cough/sneezing etiquettes. Individuals with severe symptoms and, therefore, under treatment are assumed to have a reduced rate of viral shedding rate by a factor *η*_3_ ≪ 1. The coronaviruses are known to have a limited life span on various surfaces and so it is assumed that the average time taken for them to stay on surfaces is 1/*ν* and that time can be further reduced through disinfection control 0 ≤ *u*_4_ ≤ 1.

(1) $$\begin{array}{c} \left.\begin{array}{c} \frac{\text{d}S}{\text{d}t}=\left(1-f_{1}-f_{2}\right)\Lambda +\theta R-\left(\lambda_{1}+\lambda_{2}+\mu \right)S,\\ \frac{\text{d}E}{\text{d}t}=f_{1}\Lambda +\left(\lambda_{1}+\lambda_{2}\right)S-\left(\rho +\delta_{1}+\mu \right)E,\\ \frac{\text{d}I_{1}}{\text{d}t}=f_{2}\Lambda +\delta_{1}\left(1-g\right)E-\left(\rho +\delta_{2}+\mu \right)I_{1},\\ \frac{\text{d}I_{2}}{\text{d}t}=\delta_{2}I_{1}+g\delta_{1}E-\left(\rho +\mu +\mu_{d}\right)I_{2},\\ \frac{\text{d}R}{\text{d}t}=\rho \left(E+I_{1}+I_{2}\right)-\left(\theta +\mu \right)R,\\ \frac{\text{d}V}{\text{d}t}=\xi \left(1-u_{3}\right)\left(E+I_{1}+\eta_{3}I_{2}\right)-\left(u_{4}\nu +\mu_{v}\right)V, \end{array}\right\} \end{array}$$

where the following notations are used for convenience:

(2) $$\begin{array}{c} k_{1}=\rho +\delta_{1}+\mu ,\\ k_{2}=\rho +\delta_{2}+\mu ,\\ k_{3}=\rho +\mu +\mu_{d},\\ k_{4}=\theta +\mu ,\\ k_{5}=\nu u_{4}+\mu_{v}. \end{array}$$

The parameters of the model are further summarized in Table 1.

## 3. Main Results

### 3.1. Positivity and Boundedness of Model Solution

We state the result of the positivity and boundedness of solutions of model (1) in Lemma 1.

**Lemma 1 1.**

Given Ω={(*S*, *E*, *I*_1_, *I*_2_, *R*, *V*) ∈ *ℝ*_≥0_^6^*|N* ≤ (Λ/*μ*) and *V* ≤ (*ξ*(1 − *u*_3_)Λ/*μ*(*u*_4_*ν*+*μ*_*v*_))}, all solutions of (1) starting in Ω remain in Ω for all *t* ≥ 0. Also, the region is a positively invariant set for the model (1).

**Proof**

Let *t*_1_=sup{*t* > 0*|S* ≥ 0, *E* ≥ 0, *I*_1_ ≥ 0, *I*_2_ ≥ 0, *R* ≥ 0, *V* ≥ 0} and *λ*(*t*)=*λ*_1_(*t*)+*λ*_2_(*t*).

The first equation of (1) yields

(3) $$\begin{array}{c} \frac{\text{d}S}{\text{d}t}+\left(\lambda +\mu \right)S\geq \left(1-f_{1}-f_{2}\right)\Lambda . \end{array}$$

Then,

(4) $$\begin{array}{c} \frac{\text{d}}{\text{d}t}\left[S\left(t\right)\exp\left\{\mu t+\int_{0}^{t}\lambda \left(s\right)\text{d}s\right\}\right]\geq \left(1-f_{1}-f_{2}\right)\Lambda \;\exp\left\{\mu t+\int_{0}^{t}\lambda \left(s\right)\text{d}s\right\}. \end{array}$$

Hence,

(5) $$\begin{array}{c} S\left(t_{1}\right)\exp\left\{\mu t_{1}+\int_{0}^{t_{1}}\lambda \left(s\right)\text{d}s\right\}-S\left(0\right)\geq \int_{0}^{t_{1}}\left[\left(1-f_{1}-f_{2}\right)\Lambda \;\exp\left\{\mu \nu +\int_{0}^{\nu }\lambda \left(s\right)\text{d}s\right\}\right]\text{d}\nu ,\\ S\left(t_{1}\right)\geq \;\exp\left\{-\mu t_{1}-\int_{0}^{t_{1}}\lambda \left(s\right)\text{d}s\right\}\times \left\{S\left(0\right)+\int_{0}^{t_{1}}\left[\left(1-f_{1}-f_{2}\right)\Lambda \;\exp\left\{\mu \nu +\int_{0}^{\nu }\lambda \left(s\right)\text{d}s\right\}\right]\text{d}\nu \right\}. \end{array}$$

Clearly, *S*(*t*_1_) ≥ 0.

Similar arguments can be used to show that *E* ≥ 0, *I*_1_ ≥ 0, *I*_2_ ≥ 0, *R* ≥ 0, and *V* ≥ 0, and thus, all solutions with nonnegative initial conditions are nonnegative.

Further, adding the first five subequations of (1) gives

(6) $$\begin{array}{c} \frac{\text{d}N}{\text{d}t}=\Lambda -\mu N-\mu_{d}I_{2}\leq \Lambda -\mu N. \end{array}$$

Thus, *N*(*t*) ≤ *N*(0)*e*^−*μt*^+(Λ/*μ*)(1 − *e*^−*μ*^).

Therefore, if 0 ≤ *N*(0) ≤ (Λ/*μ*), then, ≤limsup_*t*⟶*∞*_*N*(*t*) ≤ (Λ/*μ*).

The last equation of (1) implies that

(7) $$\begin{array}{c} \frac{\text{d}V}{\text{d}t}=\xi \left(1-u_{3}\right)\left(E+I_{1}+\eta_{3}I_{2}\right)-\left(u_{4}\nu +\mu_{v}\right)V\leq \frac{\xi \left(1-u_{3}\right)\Lambda }{\mu }-\left(u_{4}\nu +\mu_{v}\right)V. \end{array}$$

So if 0 ≤ *V*(0) ≤ (*ξ*(1 − *u*_3_)Λ/*μ*), then 0 ≤ *V*(*t*) ≤ (*ξ*(1 − *u*_3_)Λ/*μ*(*u*_4_*ν*+*μ*_*v*_)). Thus, all solutions starting within Ω remain inside Ω. This completes the proof of the lemma.

### 3.2. Equilibria and Basic Reproduction Number

In the presence of immigration of infective persons, the model (1) does not have a disease-free equilibrium. However, where there are no infective immigrants, the disease-free equilibrium is given by *ε*_0_=((Λ/*μ*), 0,0,0,0,0,0), and using the Next-Generation Method of [11], the basic reproduction number of the model (when *f*_1_=*f*_2_=0) is given by

(8) $$\begin{array}{c} {ℛ}_{0}=\overset{\text{Infections from infectious individuals}}{\overset{︷}{\frac{\beta c\left(1-u_{1}\right)\left[\eta_{1}k_{3}k_{2}+\delta_{1}\left(1-g\right)k_{3}+\eta_{2}\left(g\left(\rho +\mu \right)+\delta_{2}\right)\delta_{1}\right]}{k_{3}k_{2}k_{1}}}}\\ +\underset{\text{Infections from coronavirus}-\text{contaminated surfaces}}{\underset{︸}{\frac{\beta_{v}\left(1-u_{2}\right)\left(1-u_{3}\right)\xi \left[\left(\eta_{3}+1\right)\delta_{1}\left(g\left(\rho +\mu \right)+\delta_{2}\right)+k_{3}k_{2}\right]\Lambda }{\mu k_{3}k_{2}Kk_{1}k_{5}}}}. \end{array}$$

From the expression of ℛ_0_, it is noted that if the controls *u*_1_, *u*_2_, and *u*_3_ are implemented with 100% efficacy, the disease easily is eradicated. The following result follows from [11].

**Theorem 1 .**

In the absence of immigration of infective persons, the COVID-19 model (1) possesses a disease-free equilibrium (*ε*_0_) which is locally asymptotically stable whenever ℛ_0_ < 1 and unstable whenever ℛ_0_ > 1.

In the presence of infective immigrants, the model always exhibits an endemic equilibrium *ε*^*∗*^=(*S*^*∗*^, *E*^*∗*^, *I*_1_^*∗*^, *I*_2_^*∗*^, *R*^*∗*^, *V*^*∗*^), where

(9) $$\begin{array}{c} \left.\begin{array}{c} S^{*}=\frac{\theta Z_{5}E^{*}}{\lambda^{*}+\mu }+\frac{Z_{8}}{\lambda^{*}+\mu },\\ E^{*}=\frac{\Lambda \mu f_{1}+\lambda^{*}Z_{9}}{\lambda^{*}Z_{10}+\mu k_{1}},\\ I_{1}^{*}=Z_{1}E^{*}+Z_{0},\\ I_{2}^{*}=Z_{3}E^{*}+Z_{2},\\ R^{*}=Z_{5}E^{*}+Z_{4},\\ V^{*}=Z_{7}E^{*}+Z_{6}. \end{array}\right\} \end{array}$$

where *Z*_*i*_,  *i*=1,2,…, 10, are presented in Appendix.

We note that *λ*^*∗*^=*λ*_1_^*∗*^+*λ*_2_^*∗*^, which characterises the endemic equilibrium, satisfies the following equation:

(10) $$\begin{array}{c} {\mathbb{C}}_{3}{\left(\lambda^{*}\right)}^{3}+{\mathbb{C}}_{2}{\left(\lambda^{*}\right)}^{2}+{\mathbb{C}}_{1}\lambda^{*}+{\mathbb{C}}_{0}=0, \end{array}$$

where the coefficients *ℂ*_*i*_, *i*=0,…, 3, are defined in Appendix.

The existence of endemic equilibria of the COVID-19 model (1) is determined by the roots of (10) given by

(11) $$\begin{array}{c} \lambda^{*}=\sqrt[{3}]{\frac{\Delta_{1}\pm \sqrt{\Delta_{1}^{2}-4\Delta_{0}^{3}}}{2}}, \end{array}$$

where Δ_0_=*ℂ*_2_^2^ − 3*ℂ*_3_*ℂ*_1_, and Δ_1_=2*ℂ*_2_^3^ − 9*ℂ*_3_*ℂ*_2_*ℂ*_1_+27*ℂ*_3_^2^*ℂ*_0_.

In the next section, the sensitivity of the basic reproduction number ℛ_0_ and the endemic equilibrium *ε*^*∗*^ to the model parameters is discussed.

### 3.3. Sensitivity Analysis

Due to the uncertainty/inaccuracies surrounding the measurement of model parameters, it is important to study, for each model, the influence of model parameters on model predictions so that those parameters which are observed to have a greater impact are targeted and measured with more accuracy/precision. If data is unavailable for such parameters, we could then focus on getting the needed data to estimate such parameters. Since our interest is to curb the spread of COVID-19, which is possible in the absence of infective immigrants when ℛ_0_ < 1, we determine the sensitivity indexes of model parameters determining ℛ_0_ in order to identify those parameters that can be used to drive ℛ_0_ to below unity. The sensitivity index of a typical parameter *p*_*i*_ upon which ℛ_0_ differentiability depends is defined by Υ_ℛ_0__^*p*_*i*_^=(∂ℛ_0_/∂*p*_*i*_) × (*p*_*i*_/ℛ_0_)*|*_*p*_, where **p**=(*p*_1_, *p*_2_,…, *p*_*n*_) is the vector of baseline parameter values at which the sensitivity indexes are evaluated. To determine the sensitivity indexes of the endemic equilibrium, we used the technique of [12], which is summarized as follows.For the model (d*x*/d*t*)=*f*(*x*, *p*) where *x* ∈ *ℝ*^*m*^ and *p* ∈ *ℝ*^*n*^ are vectors of state variables and model parameters, respectively, the sensitivity index of a typical endemic equilibrium state variable *x*_*i*_^*∗*^ with respect to the model parameter *p*_*j*_ is given by

(12) $$\begin{array}{c} \Upsilon_{x_{i}^{*}}^{p_{j}}=-\left(J_{x^{*}}^{-1}J_{p}\right)\times \frac{p_{j}}{x_{i}^{*}}, \end{array}$$

where *𝒥*_*x*^*∗*^_ and *𝒥*_*p*_ are *m* × *m* and *m* × *n* matrices representing the Jacobian of the model with respect to state variables *x* and model parameters *p*, respectively, evaluated at the endemic equilibrium and baseline parameter values. These matrices are defined by

(13) $$\begin{array}{c} J_{x^{*}}={\frac{\partial f\left(x,p\right)}{\partial x}|}_{\left(x^{*},p\right)},\\ J_{p}={\frac{\partial f\left(x,p\right)}{\partial p}|}_{\left(x^{*},p\right)}. \end{array}$$

We note that the summary description herein of the technique of [12] makes implementation easier especially with computer algebra systems (CAS) since one only needs to define the model in the appropriate format for the CAS and identify the state variables and model parameters. The sensitivity indices of the parameters are presented in Table 2.

It can be noted from Table 2 that increasing Λ, *β*, *β*_*v*_, *c*, *ξ*, *η*_1_, *η*_2_, and *η*_3_ increases ℛ_0_ (and consequently increases *E*^*∗*^, *I*_1_^*∗*^, and *I*_2_^*∗*^) while increasing *ρ*, *δ*_1_, *mu*, *μ*_*d*_, *μ*_*v*_, and *K* decreases ℛ_0_ (and consequently decreases *E*^*∗*^, *I*_1_^*∗*^, and *I*_2_^*∗*^). Therefore, efforts aimed at reducing Λ, *β*, *β*_*v*_, *c*, *ξ*, *η*_1_, *η*_2_, and *η*_3_ and increasing *ρ*, *δ*_1_, *mu*, *μ*_*d*_, *μ*_*v*_, and *K* should be made in order to keep ℛ_0_ (and consequently, *E*^*∗*^, *I*_1_^*∗*^, and *I*_2_^*∗*^) small enough to contain or eradicate COVID-19.

### 3.4. Optimal Control of COVID-19 Spread

In order to determine the best strategy to adopt in the fight against COVID-19, optimal control theory is employed by formulating the following optimization problem:

(14) $$\begin{array}{c} \left.\begin{array}{cc} \underset{u_{i}}{\min} & J\left(u_{1},u_{2},u_{3},u_{4}\right),\\ \text{Subject to}: & \begin{array}{c} \frac{\text{d}S}{\text{d}t}=\left(1-f_{1}-f_{2}\right)\Lambda +\theta R-\left(\lambda_{1}+\lambda_{2}+\mu \right)S,\\ \frac{\text{d}E}{\text{d}t}=f_{1}\Lambda +\left(\lambda_{1}+\lambda_{2}\right)S-\left(\rho +\delta_{1}+\mu \right)E,\\ \frac{\text{d}I_{1}}{\text{d}t}=f_{2}\Lambda +\delta_{1}\left(1-g\right)E-\left(\rho +\delta_{2}+\mu \right)I_{1},\\ \frac{\text{d}I_{2}}{\text{d}t}=\delta_{2}I_{1}+g\delta_{1}E-\left(\rho +\mu +\mu_{d}\right)I_{2},\\ \frac{\text{d}R}{\text{d}t}=\rho \left(E+I_{1}+I_{2}\right)-\left(\theta +\mu \right)R,\\ \frac{\text{d}V}{\text{d}t}=\xi \left(1-u_{3}\right)\left(E+I_{1}+\eta_{3}I_{2}\right)-\left(u_{4}\nu +\mu_{v}\right)V, \end{array} \end{array}\right\} \end{array}$$

where

(15) $$\begin{array}{c} J\left(u_{1},u_{2},u_{3},u_{4}\right)=\int_{t_{0}}^{t_{f}}\left(A_{1}E+A_{2}I_{1}+A_{3}I_{2}+A_{4}V+\sum_{i=1}^{4}B_{i}u_{i}^{2}\right)\text{d}t. \end{array}$$

The objective functional *J*(*u*_1_, *u*_2_, *u*_3_, *u*_4_) measures the total infections and total costs associated with the controls. The coefficients *A*_*i*_,  ∀*i*=1,2,…, 4 are balancing factors accounting for the differences in the importance of the state variables and controls in *J*. The goal here is to seek an optimal quadruple (*u*_1_^*∗*^, *u*_2_^*∗*^, *u*_3_^*∗*^, *u*_4_^*∗*^) that minimizes the functional *J*, where *𝒰* is the set of all admissible controls. The Pontryagin maximum principle [13] provides the necessary optimality conditions for the optimal tuple. Pontryagin's principle converts the problem of minimizing *J* subject to the equations in (1) into a problem of pointwise minimization of a Hamiltonian given by

(16) $$\begin{array}{c} ℋ=\frac{\text{d}J}{dt}+\sum_{i}M_{i}\frac{\text{d}i}{\text{d}t},\;\forall i\in \left\{S,E,I_{1},I_{2},R,V\right\}, \end{array}$$

where *M*_*S*_, *M*_*E*_, *M*_*I*_1__, *M*_*I*_2__, *M*_*R*_, and *M*_*V*_ are the adjoint variables associated with the state variables. The following result is easy to establish.

**Theorem 2 .**

Let (*S*^*∗*^, *E*^*∗*^, *I*_1_^*∗*^, *I*_2_^*∗*^, *R*^*∗*^, *V*^*∗*^) be the solution associated with the tuple (*u*_1_^*∗*^, *u*_2_^*∗*^, *u*_3_^*∗*^, *u*_4_^*∗*^) that minimizes *J* over *𝒰*. Then,

1. There exists adjoint variables *M*_*x*_ such that

(17) $$\begin{array}{c} \frac{\text{d}M_{x}}{\text{d}t}=-\frac{\text{d}H}{\text{d}x},\;\forall x\in \left\{S,E,I_{1},I_{2},R,V\right\}. \end{array}$$

(2) The following transversality conditions hold:

(18) $$\begin{array}{c} M_{x}\left(t_{f}\right)=0,\;\forall x\in \left\{S,E,I_{1},I_{2},R,V\right\}. \end{array}$$

(3) $u_{i}^{*}=\max\left\{0,\min\left\{1,\tilde{u_{i}}\right\}\right\}$, where

(19) $$\begin{array}{c} \left.\begin{array}{c} \tilde{u_{1}}=\frac{\beta c\left(M_{E}-M_{S}\right)\left(\eta_{1}E+\eta_{2}I_{2}+I_{1}\right)S}{2B_{1}\left[N-q\left(E+I_{1}+I_{2}\right)\right]},\\ \tilde{u_{2}}=\frac{VS\beta_{v}\left(M_{E}-M_{S}\right)}{2\left(K+V\right)B_{2}},\\ \tilde{u_{3}}=\frac{M_{V}\xi \left(I_{2}\eta_{3}+E+I_{2}\right)}{2B_{3}},\\ \tilde{u_{4}}=\frac{\nu M_{V}V}{2B_{4}}. \end{array}\right\} \end{array}$$

**Proof**

Corollary 4.1 of [14] shows the existence of an optimal quadrupole due to the convexity of the integrand of *J* with the controls, a priori boundedness of the state solutions, and the Lipschitz property of the state system with respect to the state variables. The equations in (17) governing the adjoint variables are obtained by differentiation of the Hamiltonian with respect to the associated state variables evaluated at the optimal control.

The expressions for $\tilde{u_{i}}$ are obtained from d*H*/d*u*_*i*_=0, which hold at optimality. Using standard control arguments involving the bounds on the controls gives the characterisations for the controls in (19).

## 4. Numerical Experimentation

In this section, some numerical experiments are performed, first to study the impact of the various model parameters on the spread on COVID-19 and to illustrate the analytical results obtained and, second, via the optimal control, to determine the best strategy that can be used to combat COVID-19 spread. All simulations are done using the parameter values in Table 1.

### 4.1. Simulation with Constant Controls

**Figure 1:**
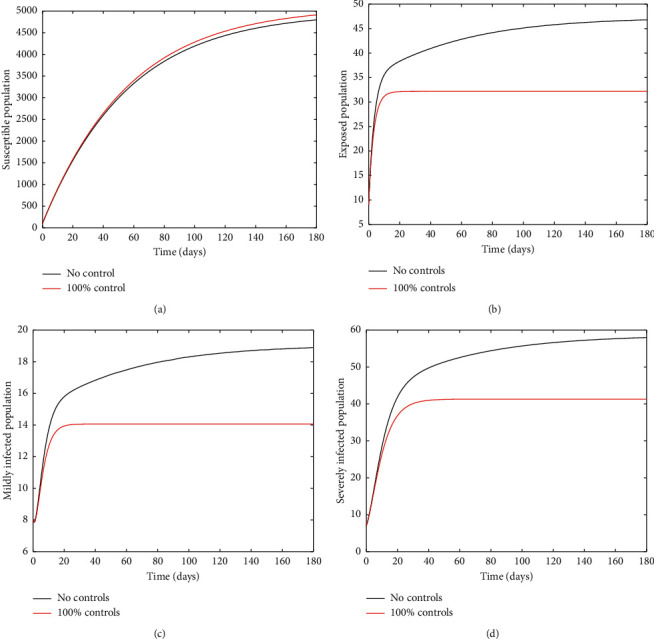
Time series of *S*, *E*, *I*_1_, and *I*_2_ for 0% and 100% implementation of controls (in this case, ℛ_0_=0.9586).

Figure 1 compares results of the simulation for the case of no controls at all on the one hand (i.e., 0% implementation of the controls) and where all four controls are implemented to perfection (i.e., 100% implementation) on the other hand. It is observed that even though implementing the controls to perfection produces some desirable results as it leads to reduced (and stabilized) infections, the disease persists during the whole period of study even with ℛ_0_ < 1. This is attributable to the fact that immigrants are still allowed into the country.

**Figure 2:**
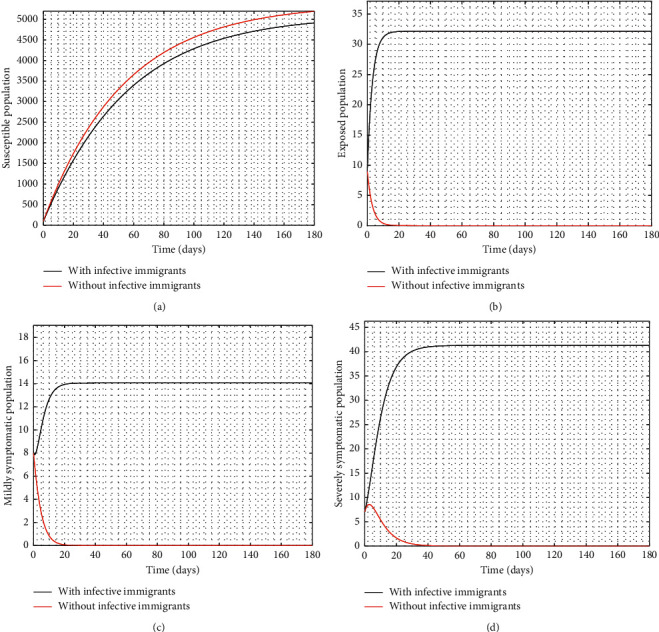
Time series of *S*, *E*, *I*_1_, and *I*_2_ at 100% implementation of controls with and without infective immigrants (ℛ_0_=0.9586).

Figure 2 compares the simulation at 100% implementation of all controls with and without infective immigrants.

From Figure 2, it is observed that with effective border closure, if the necessary actions are taken to detect and treat exposed and symptomatic persons, COVID-19 can be eradicated within the first month of implementation of the strategies. It is also observed that a perfect implementation of all controls is not sufficient to stop the spread of COVID-19 unless there is a restriction on immigration. Therefore, the decision by governments all over the world to close their borders was in the right direction.

What happens if, after some time, the government decides to allow for unrestricted immigration? To illustrate this scenario, the fractions of immigrants who are exposed *f*_1_ and those who are mildly symptomatic *f*_2_ are reformulated as follows:

(20) $$\begin{array}{c} f_{1}=\left\{\begin{array}{cc} 0, & t\leq T,\\ >0, & T<t\leq T_{f}, \end{array}\right.\\ f_{2}=\left\{\begin{array}{cc} 0, & t\leq T,\\ >0, & T<t\leq T_{f}. \end{array}\right. \end{array}$$

**Figure 3:**
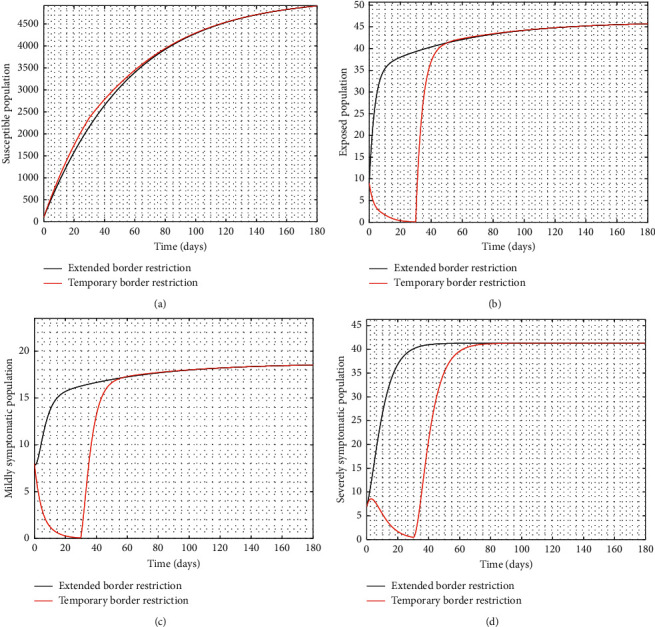
Time series of *S*, *E*, *I*_1_, and *I*_2_ at 100% implementation of controls with extended and temporary border closure (ℛ_0_=0.9586).

Experimenting with *T*=30 days, the result of the simulation is presented in Figure 3.

It is observed from Figure 3 that even after the disease is eradicated, a relaxation of the borders to allow immigration of all manner of persons will trigger a second wave of the disease. Therefore, the need to continue to close borders or screen immigrants in order to identify, quarantine, and treat infected persons cannot be overemphasized even after the community transmissions are eliminated.

### 4.2. Simulation of Optimal Control

The question of which combination of the four controls (*u*_1_, *u*_2_, *u*_3_, and *u*_4_) is most cost-effective in combatting the spread of SARS-CoV-2 is considered here. The optimal control problem (14) is solved for all possible combinations of the controls to determine which combination is most cost-effective. The optimal control problem (5) can be solved as a boundary value problem consisting of the state system (1) endowed with initial conditions and the adjoint system (17) endowed with final conditions (18). We make use of the bvp4c function in MATLAB to solve the boundary value problem for each of the fifteen (15) combinations of the controls (see Table 3) calculating (see Table 4) the total number averted of exposed persons *E*, mildly symptomatic *I*_1_, severely symptomatic *I*_2_, surface concentration of SARS-CoV-2 *V*, *J*_2_^*∗a*^=*E*+*I*_1_+*I*_2_, the weighted sum *J*_2_^*∗b*^=*A*_1_*E*+*A*_2_*I*_1_+*A*_3_*I*_2_+*A*_4_*V*, and the total cost incurred in implementing the control set. The total infections averted are the difference between the total infections without controls (*u*_*i*_=0,  ∀*i*) and the total infections with control.

For each of the outputs *E*, *I*_1_, *I*_2_, *V*, *J*_2_^*∗a*^, and *J*_2_^*∗b*^, the control strategies are ranked (see Table 5) from the most cost-effective to the least effective in minimizing the output, using incremental cost-effectiveness ratio (ICER). The ICER is used to compare the cost and benefits of two competing strategies and is defined as follows.

Let the benefits of strategies *A* and *B* be *A*_*b*_ and *B*_*b*_, respectively, and let the associated costs be *A*_*c*_ and *B*_*c*_, respectively. The cost-effectiveness ratio for strategies *A* and *B* is determined as follows:

(21) $$\begin{array}{c} \text{ICER }\left(A\right)=\frac{A_{b}}{A_{c}},\\ \text{ICER }\left(B\right)=\frac{B_{b}-A_{b}}{B_{c}-A_{c}}, \end{array}$$

where the reference strategy *A* is chosen as the one with the least benefit. The strategy with the least ICER value is said to be the most cost-effective.

It is observed that the most cost-effective strategy in minimizing infections only (without environmental contamination) is strategy 8 which consists of implementing only control *u*_1_, and if minimizing environmental contamination is also included, the most cost-effective strategy is 10, which involves implementing only controls *u*_2_ and *u*_3_. We note, however, that person-to-person infection continues to be the main source of infections and, hence, strategy 8 is recommended. That is, all efforts (physical distancing, wearing of nose masks among others) aimed at reducing/preventing person-to-person transmission should be adhered to strictly.

## 5. Conclusions

In this paper, a deterministic mathematical model has been proposed to study the dynamics of the COVID-19 in a variable-sized population. It is shown that unless the immigration of infected persons is restricted, the disease cannot be eradicated, and that if border closure is strictly adhered to, the disease can be eradicated if a threshold parameter ℛ_0_ is kept below unity. The model is modified into an optimal control problem by seeking to minimize an objective function that measures total infected persons and surface viral concentration and the total cost associated with implementing the various controls. It is shown that the best strategy in controlling the spread of COVID-19 is social distancing and use of nose masks.

## Figures and Tables

**Table 1:** Description of model parameters and baseline values.

Par	Description	Value	Source
Λ	Recruitment rate	1000	Assumed
*β*	Probability of infection per contact	8.073×10^−3^	[5]
*c*	Average contacts of infectious person per time	0.5297	[9]
*θ*	Rate of loss of immunity after recovery	0.0357	—
*ρ*	Rate of recovery from COVID-19	1/10	[5]
*δ*_1_	Rate of progression from exposure to the symptomatic stage	1/5.2	[10]
*δ*_2_	Rate of progression from mild to severe symptomatic stage	1/5.8	[10]
*μ*	Natural death rate in humans	1.86×10^−2^	—
*μ*_*d*_	COVID-19-induced death rate in humans	1.50×10^−2^	[5]
*μ*_*v*_	Death rate of coronaviruses on surfaces	3.33×10^−1^	Estimated
*f*_1_	Proportion of immigrants who are exposed	0.100	—
*f*_1_	Proportion of immigrants who are mildly symptomatic	0.0100	—
*η*_1_	Coefficient of infectivity of exposed persons	1.5	Assumed
*η*_2_	Coefficient of infectivity of severely symptomatic persons	0.100	Assumed
*η*_3_	Coefficient of viral shedding of severely symptomatic persons	0.001	Assumed
*q*	Efficacy of quarantine to prevent transmission	0.5	Assumed
*β*_*v*_	Surface-to-human transmission probability	0.001	—
*K*	Coronavirus concentration on surfaces	10^3^ cells/m^2^	—
*ξ*	Viral shedding rate of infected persons	100 cells/day	—
*ν*	Rate of disinfection of the environment	—	—

**Table 2:** Sensitivity indexes of ℛ_0_ and *ε*^*∗*^ with respect to model parameters.

Par.	Output variables						
	ℛ_0_	*S* ^*∗*^	*E* ^*∗*^	*I*_1_^*∗*^	*I*_2_^*∗*^	*R* ^*∗*^	*V* ^*∗*^
Λ	0.8946	0.9884	1.1660	1.1350	1.1530	1.1550	1.1570
*f*_1_	0.0000	−0.06107	0.8750	0.7072	0.8040	0.8158	0.8263
*f*_2_	0.0000	−0.005847	0.00946	0.1994	0.08979	0.07641	0.0646
*β*	0.1054	−0.008432	0.1208	0.0976	0.111	0.1126	0.1141
*β*_*v*_	0.8946	−0.01796	0.2573	0.2080	0.2365	0.2400	0.2430
*C*	0.1054	−0.008432	0.1208	0.09764	0.111	0.1126	0.1141
*g*	0.106	0.001847	−0.04294	−0.843	0.1957	−0.054	−0.2747
*ρ*	−0.7521	0.02509	−0.4128	−0.6773	−1.273	0.1424	−0.4903
*δ*_1_	−0.1164	0.004036	−0.7009	0.2417	0.2748	−0.09762	−0.4268
*δ*_2_	0.06281	0.001354	−0.03147	−0.6179	0.1435	−0.03958	−0.2013
*μ*	−1.0340	−1.0200	−0.3479	−0.3451	−0.4859	−0.7548	−0.3472
*μ*_*d*_	−0.05436	−0.004219	−0.001719	−0.001389	−0.1139	−0.05433	−0.0017
*ν*	−0.2066	0.001709	−0.02448	−0.01979	−0.0225	−0.02283	−0.2541
*μ*_*v*_	−0.688	0.00569	−0.08152	−0.06589	−0.07491	−0.07601	−0.846
*ξ*	0.8946	−0.007399	0.106	0.08568	0.09741	0.09884	1.1000
*θ*	0.0000	0.02745	0.01066	0.008617	0.009797	−0.6475	0.01007
*Q*	0.0000	−0.0001883	0.002697	0.00218	0.002479	0.002515	0.0025
*η*_1_	0.08128	−0.00622	0.08911	0.07203	0.08189	0.08309	0.08415
*η*_2_	0.006211	−0.0005172	0.00741	0.005989	0.006809	0.006909	0.006997
*η*_3_	4.775*e* − 4	−6.545*e* − 6	9.377*e* − 5	7.579*e* − 5	8.616*e* − 5	8.743*e* − 5	9.731*e* − 4
*K*	−0.8946	0.007399	−0.106	−0.08568	−0.09741	−0.09884	−0.1001

**Table 3:** List of all strategies used for the simulation of the optimal control problem.

Strategy	Control combination
1	(*u*_1_ ≠ 0, *u*_2_ ≠ 0, *u*_3_ ≠ 0, *u*_4_ ≠ 0)
2	(*u*_1_ ≠ 0, *u*_2_ ≠ 0, *u*_3_ ≠ 0, *u*_4_=0)
3	(*u*_1_ ≠ 0, *u*_2_ ≠ 0, *u*_3_=0, *u*_4_ ≠ 0)
4	(*u*_1_ ≠ 0, *u*_2_ ≠ 0, *u*_3_=0, *u*_4_=0)
5	(*u*_1_ ≠ 0, *u*_2_=0, *u*_3_ ≠ 0, *u*_4_ ≠ 0)
6	(*u*_1_ ≠ 0, *u*_2_=0, *u*_3_ ≠ 0, *u*_4_=0)
7	(*u*_1_ ≠ 0, *u*_2_=0, *u*_3_=0, *u*_4_ ≠ 0)
8	(*u*_1_ ≠ 0, *u*_2_=0, *u*_3_=0, *u*_4_=0)
9	(*u*_1_=0, *u*_2_ ≠ 0, *u*_3_ ≠ 0, *u*_4_ ≠ 0)
10	(*u*_1_=0, *u*_2_ ≠ 0, *u*_3_ ≠ 0, *u*_4_=0)
11	(*u*_1_=0, *u*_2_ ≠ 0, *u*_3_=0, *u*_4_ ≠ 0)
12	(*u*_1_=0, *u*_2_ ≠ 0, *u*_3_=0, *u*_4_=0)
13	(*u*_1_=0, *u*_2_=0, *u*_3_ ≠ 0, *u*_4_ ≠ 0)
14	(*u*_1_=0, *u*_2_=0, *u*_3_ ≠ 0, *u*_4_=0)
15	(*u*_1_=0, *u*_2_=0, *u*_3_=0, *u*_4_ ≠ 0)

**Table 4:** Results from the simulation of the optimal control problem.

Strategy	Total cost/infections averted						
	*E*	*I*_1_	*I*_2_	*J*_2_^*∗a*^	*V*	*J*_2_^*∗b*^	Cost (*C*)
1	2611.359	993.9245	3089.437	6694.72	1428001	24083.05	506.7692
2	2733.639	1047.799	3235.659	7017.097	1289513	23169.77	409.1593
3	1764.43	629.6668	2014.547	4408.643	2062500	27112.9	1711.077
4	−5760.75	−2641.78	−7402.5	−15805	−6571334	−88600	4028.777
5	2570.829	978.5093	3047.142	6596.481	1714556	26802.12	512.8923
6	2666.764	1018.451	3143.797	6829.012	1206425	22069.25	412.3039
7	936.6537	340.6382	1059.084	2336.376	1727553	20718.88	1006.99
8	1386.963	531.5784	1605.459	3524	1283545	18012.2	222.5826
9	2548.109	987.5465	3006.603	6542.258	3394931	43533.45	315.2923
10	2546.287	985.938	2985.105	6517.33	3155681	41113.4	266.7208
11	2733.361	1070.782	3275.858	7080.001	2332750	33676.25	820.8051
12	2595.299	1010.626	3084.501	6690.426	1580560	25596.63	475.1345
13	2616.866	1016.852	3088.199	6721.918	3412642	43973.63	299.1677
14	2685.096	1044.349	3147.14	6876.585	3216295	42246.81	240.0045
15	2962.699	1192.866	3585.314	7740.879	2692650	38226.51	285.9898

**Table 5:** Ranking of strategies from most to least cost-effective for various outputs.

Output used for ranking					
*E*	*I*_1_	*I*_2_	*J*_2_^*∗a*^	*V*	*J*_2_^*∗b*^
8	8	8	8	10	10
7	7	7	7	14	14
12	15	15	15	13	13
15	12	12	12	9	9
4	4	4	4	15	15
2	10	2	2	7	7
10	2	6	6	8	8
6	6	1	10	12	12
1	3	10	11	11	11
5	1	5	5	3	3
3	14	3	3	4	4
14	13	14	14	6	6
13	9	13	13	2	2
9	5	9	9	1	1
11	11	11	11	5	5

## Data Availability

All data used to support the findings of this study are included within the article.

## Appendix

### Coefficients of *λ*^*∗*^ in Equation (10)

(A.1) $$\begin{array}{c} {\mathbb{C}}_{3}=-\left[\left(K+Z_{6}\right)Z_{10}+Z_{7}Z_{9}\right]\left[\left(q\left(Z_{0}+Z_{2}\right)\mu +Z_{2}\mu_{d}-\Lambda \right)Z_{10}+Z_{9}\left(q\left(Z_{1}+Z_{3}+1\right)\mu +\mu_{d}Z_{3}\right)\right], \end{array}$$

(A.2) $$\begin{array}{c} C_{2}=\left[\left(-\left(Z_{0}+Z_{2}\right)\left(\left(K+Z_{6}\right)Z_{10}+Z_{7}Z_{9}\right)-\left(\left(Z_{0}+Z_{2}\right)Z_{10}+Z_{9}\left(Z_{1}+Z_{3}+1\right)\right)\left(K+Z_{6}\right)\right)k_{1}\right.\\ +\left[\left(-Z_{2}\left(\left(K+Z_{6}\right)Z_{10}+Z_{7}Z_{9}\right)+\left(-Z_{2}Z_{10}-Z_{3}Z_{9}\right)\left(K+Z_{6}\right)\right)k_{1}\right.\\ \left.-\Lambda Z_{3}f_{1}\left(\left(K+Z_{6}\right)Z_{10}+Z_{7}Z_{9}\right)+\left(-Z_{2}Z_{10}-Z_{3}Z_{9}\right)\Lambda Z_{7}f_{1}\right]\mu \mu_{d}\\ +\left[\left(\Lambda \left(\left(K+Z_{6}\right)Z_{10}+Z_{7}Z_{9}\right)+\Lambda Z_{10}\left(K+Z_{6}\right)\right)k_{1}+\Lambda^{2}Z_{10}Z_{7}f_{1}\right]\mu \\ -\beta c\mu \left(1-u_{1}\right)\left(Z_{9}\left(Z_{3}\eta_{2}+Z_{1}+\eta_{1}\right)+\left(Z_{2}\eta_{2}+Z_{0}\right)Z_{10}\right)\left(\left(K+Z_{6}\right)Z_{10}+Z_{7}Z_{9}\right)\\ +\beta_{v}\left(1-u_{2}\right)\left(Z_{6}Z_{10}+Z_{7}Z_{9}\right)\left[q\mu \left(\left(Z_{0}+Z_{2}\right)Z_{10}+Z_{9}\left(Z_{1}+Z_{3}+1\right)\right)+\left(Z_{2}\mu_{d}-\Lambda \right)Z_{10}+Z_{3}\mu_{d}Z_{9}\right], \end{array}$$

(A.3) $$\begin{array}{c} {\mathbb{C}}_{1}=\left[-q\left(\Lambda \left(Z_{1}+Z_{3}+1\right)f_{1}+k_{1}\left(Z_{0}+Z_{2}\right)\right)\mu -\left(Z_{2}\mu_{d}-\Lambda \right)k_{1}-Z_{3}\mu_{d}\Lambda f_{1}\right]\mu^{2}\left(\left(K+Z_{6}\right)k_{1}+\Lambda Z_{7}f_{1}\right)\\ -\beta c\mu^{2}\left(1-u_{1}\right)\left[\left(\Lambda f_{1}\left(Z_{3}\eta_{2}+Z_{1}+\eta_{1}\right)+\left(Z_{2}\eta_{2}+Z_{0}\right)k_{1}\right)\left(\left(K+Z_{6}\right)Z_{10}+Z_{7}Z_{9}\right)\right.\\ \left.+\left(Z_{9}\left(Z_{3}\eta_{2}+Z_{1}+\eta_{1}\right)+\left(Z_{2}\eta_{2}+Z_{0}\right)Z_{10}\right)\left(\left(K+Z_{6}\right)k_{1}+\Lambda Z_{7}f_{1}\right)\right]\\ -\beta_{v}\left(1-u_{2}\right)\left\{\left[-\left(\Lambda Z_{7}f_{1}+Z_{6}k_{1}\right)\left(\left(Z_{0}+Z_{2}\right)Z_{10}+Z_{9}\left(Z_{1}+Z_{3}+1\right)\right)\right.\right.\\ \left.-\left(Z_{6}Z_{10}+Z_{7}Z_{9}\right)\left(\Lambda \left(Z_{1}+Z_{3}+1\right)f_{1}+k_{1}\left(Z_{0}+Z_{2}\right)\right)\right]\mu^{2}q\\ +\left[\left(\left(\Lambda Z_{7}f_{1}+Z_{6}k_{1}\right)\left(-Z_{2}Z_{10}-Z_{3}Z_{9}\right)+\left(Z_{6}Z_{10}+Z_{7}Z_{9}\right)\left(-\Lambda Z_{3}f_{1}-Z_{2}k_{1}\right)\right)\mu_{d}\right.\\ \left.\left.+\left(\Lambda Z_{7}f_{1}+Z_{6}k_{1}\right)\Lambda Z_{10}+\left(Z_{6}Z_{10}+Z_{7}Z_{9}\right)\Lambda k_{1}\right]\mu \right\}, \end{array}$$

(A.4) $$\begin{array}{c} {\mathbb{C}}_{0}=\beta_{v}\left(1-u_{2}\right)\left[\Lambda Z_{7}f_{1}+Z_{6}k_{1}\right]\left[\left(qf_{1}\left(Z_{1}+Z_{3}+1\right)\mu +f_{1}\mu_{d}Z_{3}-k_{1}\right)\Lambda +k_{1}\left(q\left(Z_{0}+Z_{2}\right)\mu +Z_{2}\mu_{d}\right)\right]\mu^{2}\\ -\beta c\left(1-u_{1}\right)\left(\left(K+Z_{6}\right)k_{1}+\Lambda Z_{7}f_{1}\right)\left(\Lambda f_{1}\left(Z_{3}\eta_{2}+Z_{1}+\eta_{1}\right)+\left(Z_{2}\eta_{2}+Z_{0}\right)k_{1}\right)\mu^{3}, \end{array}$$

(A.5) $$\begin{array}{c} Z_{0}=\frac{f_{2}\Lambda }{k_{2}},\\ Z_{1}=\frac{\delta_{1}\left(1-g\right)}{k_{2}},\\ Z_{2}=\frac{Z_{0}\delta_{2}}{k_{3}},\\ Z_{3}=\frac{g\delta_{1}+\delta_{2}Z_{1}}{k_{3}},\\ Z_{4}=\frac{\rho \left(Z_{0}+Z_{2}\right)}{k_{4}},\\ Z_{5}=\frac{\rho \left(Z_{1}+Z_{3}+1\right)}{k_{4}},\\ Z_{6}=\frac{\xi \left(1-u_{3}\right)Z_{2}\left(\eta_{3}+1\right)}{k_{5}},\\ Z_{7}=\frac{\xi \left(1-u_{3}\right)\left(Z_{3}\eta_{3}+Z_{3}+1\right)}{k_{5}},\\ Z_{8}=\left(1-f_{1}-f_{2}\right)\Lambda +\theta Z_{4},\\ Z_{9}=f_{1}\Lambda +Z_{8},\\ Z_{10}=-\theta Z_{5}+k_{1}. \end{array}$$
